# Association of Lipoprotein-Associated Phospholipase A2 with the Prevalence of Nonalcoholic Fatty Liver Disease: A Result from the APAC Study

**DOI:** 10.1038/s41598-018-28494-8

**Published:** 2018-07-04

**Authors:** Zhongni Liu, Hong Li, Yinghong Zheng, Ziyu Gao, Lin Cong, Liming Yang, Yong Zhou

**Affiliations:** 1 Department of Pathophysiology, Key Laboratory of Cardiovascular Pathophysiology, Harbin Medical University, Harbin, China; 2 Beijing Institute of Heart, Lung and Blood Vessel Disease, Beijing Anzhen Hospital, Capital Medical University, Beijing, China

## Abstract

Nonalcoholic fatty liver disease (NAFLD) is a worldwide chronic liver disease. Few studies have investigated the association between NAFLD and Lipoprotein-associated phospholipase A_2_ (Lp-PLA_2_), a unique enzyme correlated with oxidative stress. The aim of this study was to assess the relationship between Lp-PLA_2_ and NAFLD in a Chinese community-based cohort. A total of 1587 adults aged ≥40 years were enrolled in the current study. Participants underwent a standardized evaluation. The serum Lp-PLA2 concentration was measured by ELISA and NAFLD was diagnosed by ultrasonography. Multivariable logistic regression was used to assess the association between Lp-PLA2 and NAFLD. Increased Lp-PLA2 levels were significantly associated with decreased NAFLD prevalence after adjusting for other potential confounders. The adjusted ORs of NAFLD in Q2, Q3 and Q4 compared with Q1 were 0.88 (0.64–1.21), 0.71 (0.51–0.98) and 0.67 (0.48–0.95), respectively (P < 0.05). Furthermore, the adjusted ORs of moderate and heavy NAFLD in Q2, Q3 and Q4 compared to Q1 were 0.64 (0.41–1.01), 0.48 (0.29–0.80) and 0.47 (0.28–0.79), respectively (P < 0.01). In conclusions, increased Lp-PLA2 levels were independently associated with decreased NAFLD prevalence.

## Introduction

Nonalcoholic fatty liver disease (NAFLD), one of the most common causes of chronic liver disease, is now regarded as a manifestation of metabolic syndrome with abnormal lipid deposition in liver^1,2^. Currently, the prevalence of NAFLD approaches 30% in the general population worldwide^3^. An increasing number of studies have indicated that NAFLD is linked to increased risk of cardiovascular disease (CVD)^4–6^. Meanwhile, some studies have demonstrated that lipoprotein-associated phospholipase A2 (Lp-PLA_2_), which was named firstly as platelet-activating factor acetylhydrolase (PAF-AH), is significantly associated with CVD^7,8^.

Oxidative stress is regarded as a major leading cause of NAFLD^9,10^, and the anti-oxidative property of Lp-PLA_2_ has been well addressed by several studies^11,12^. However, few studies were performed to explore the relationship between Lp-PLA_2_ and NAFLD occurrence. Previous studies have mainly focused on small, foreign populations^13,14^, but no studies have investigated this association specifically in a Chinese population.

Therefore, we hypothesized that an association between Lp-PLA_2_ and NAFLD also exists in Chinese populations. In this study, we aimed to investigate the association between serum Lp-PLA_2_ levels and NAFLD occurrence in a large Chinese community.

## Results

### Baseline characteristics

A total of 1587 participants (68.8% men) were included in the final analysis. According to the Lp-PLA_2_ quartiles, the baseline characteristics of participants are shown in Table 1. The mean Lp-PLA_2_ concentrations from the lowest to the highest quartile were 128.1 ± 2.8 ng/mL, 136.6 ± 2.7 ng/mL, 149 ± 5.1 ng/mL, and 227.3 ± 96.8 ng/mL, respectively. Age, BMI, smoking, education level, ALT, hypertension, hyperlipidaemia, triglyceride and HDL were significantly different among the participants. The mean age, HDL values and the percentage of participants with a low education level, low BMI, hypertension increased across the Lp-PLA_2_ quartiles. In contrast, the mean ALT, triglyceride levels and percentage of participants who were currently smoking and had hyperlipidaemia decreased as the Lp-PLA_2_ level increased.

**Table 1:** Baseline characteristics according to Lp-PLA_2_ quartiles.

	Total	Lp-PLA_2_ quartiles				*P-value*
		Q1	Q2	Q3	Q4	
Overall (n)	1587	396	397	397	397	
Lp-PLA_2_ mass (ng/mL)	160.3 ± 62.5	128.1 ± 2.8	136.6 ± 2.7	149 ± 5.1	227.3 ± 96.8	
Men (n, %)	1092(68.8)	278(70.2)	274(69.0)	261(65.7)	279(70.3)	0.47
Age (years)	61.6 ± 11.8	56.5 ± 9.1	59.6 ± 10.6	62.9 ± 11.7	67.3 ± 12.8	<0.01
BMI (kg/m^2^)						<0.01
<25	845(53.25)	185(46.72)	204(51.39)	222(55.92)	234(58.94)	
25–30	648(40.83)	185(46.72)	160(40.30)	158(39.80)	145(36.52)	
>30	94(5.92)	26(6.57)	33(8.31)	17(4.28)	18(4.53)	
Current smoking (n, %)	496(31.3)	154(38.9)	126(31.7)	111(28.0)	105(26.4)	<0.01
Physical activity (n, %)						0.91
Inactive	564(35.5)	142(35.9)	141(35.5)	137(34.5)	144(36.3)	
Moderate	358(22.6)	95(24.0)	90(22.7)	83(20.9)	90(22.7)	
Active	665(41.9)	159(40.2)	166(41.8)	177(44.6)	163(41.1)	
Education level (n, %)						<0.01
Illiterate or primary	310(19.5)	67(16.9)	63(15.9)	85(21.4)	95(23.9)	
Middle or high school	686(43.2)	187(47.2)	191(48.1)	155(39.0)	153(38.5)	
College or above	591(37.2)	142(35.9)	143(36.0)	157(39.5)	149(37.5)	
ALT (U/L)	17.7 ± 10.7	18.9 ± 10.3	18.6 ± 10.8	17.6 ± 11.6	15.8 ± 9.9	<0.01
Diabetes (n, %)	259(16.3)	60(15.2)	61(15.4)	75(18.9)	63(15.9)	0.45
Hypertension (n, %)	910(57.3)	198(50.0)	211(53.1)	241(60.7)	260(65.5)	<0.01
Hyperlipidaemia (n, %)	839(52.9)	231(58.3)	213(53.7)	209(52.6)	186(46.9)	0.01
Triglyceride (mmol/L)	1.6 ± 1.3	1.9 ± 1.7	1.6 ± 1.0	1.5 ± 1.0	1.4 ± 1.2	< 0.01
Total cholesterol (mmol/L)	5.2 ± 1.1	5.3 ± 1.2	5.1 ± 1.0	5.1 ± 1.0	5.1 ± 1.1	0.29
HDL (mmol/L)	1.6 ± 0.5	1.6 ± 0.7	1.5 ± 0.4	1.6 ± 0.5	1.7 ± 0.5	0.02
LDL (mmol/L)	2.7 ± 0.8	2.8 ± 0.9	2.7 ± 0.7	2.6 ± 0.7	2.6 ± 1.0	0.06
NAFLD (n, %)	650(41.0)	190(48.0%)	174(43.8)	150(37.8)	136(34.3)	<0.01

Data are expressed as the means ± SD or n (%). Abbreviation: NAFLD = nonalcoholic fatty liver disease; BMI = body mass index; ALT = alanine aminotransferase; TG = triglyceride; TC = total cholesterol; HDL = high-density lipoprotein cholesterol; LDL = low-density lipoprotein cholesterol.

### NAFLD prevalence according to the Lp-PLA_2_ quartiles

**Figure 1:**
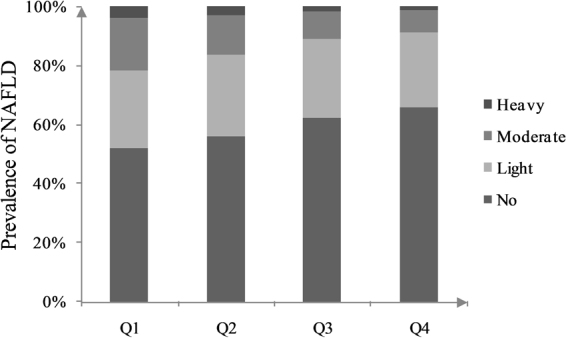
Prevalence of NAFLD according to Lp-PLA2 quartiles. No = No NAFLD; Light = Light NAFLD; Moderate = Moderate NAFLD; Heavy = Heavy NAFLD.

In our study, 41.0% (650/1587) participants were diagnosed with NAFLD. Among these participants, 26.4% (419/1587) presented with light NAFLD, 12.1% (192/1587) with moderate NAFLD and 2.5% (39/1587) with heavy NAFLD. The prevalence of no NAFLD was positively associated with Lp-PLA_2_ quartiles, whereas the prevalence of moderate and heavy NAFLD was negatively associated with Lp-PLA_2_ quartiles (Fig. 1).

### Correlation between Lp-PLA_2_ level and NAFLD

**Figure 2:**
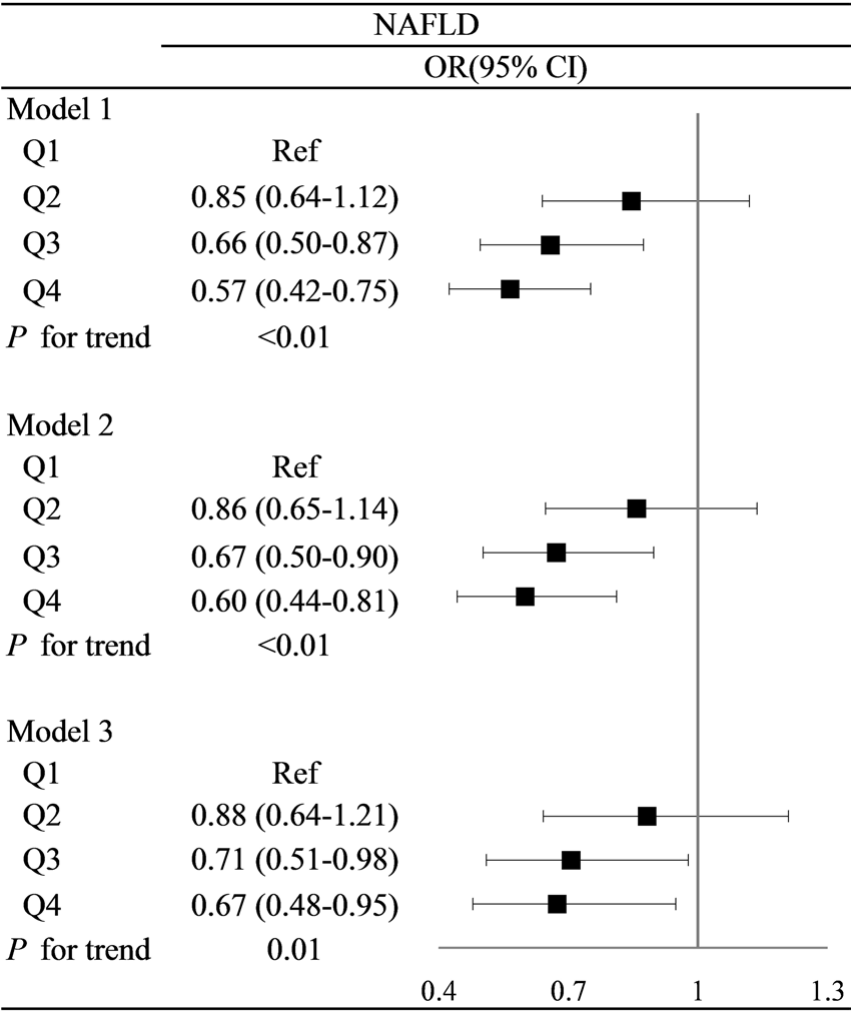
Multivariate-adjusted OR and 95% CI for NAFLD according to Lp-PLA2 quartiles. *P < 0.05. OR: odd ratio; CI: confidence interval. The Q1 group was regarded as a reference. Model 1: unadjusted. Model 2: adjusted for age and sex. Model 3: adjusted for age, sex, BMI, physical activity, education level, smoking, ALT, diabetes, hypertension and hyperlipidaemia.

Figure 2 shows the cross-sectional relationship between Lp-PLA_2_ concentration and the NAFLD prevalence. Comparing to the Q1, the ORs (95% CI) of NAFLD in Q2, Q3 and Q4 for model 1 were 0.85 (0.64–1.12), 0.66 (0.50–0.87) and 0.57 (0.42–0.75), respectively. The model 2 result, which adjusted for age and sex, was still consistent with model 1 (P < 0.01). In model 3, which adjusted for age, sex, BMI, physical activity, education level, smoking, ALT, diabetes, hypertension, hyperlipidaemia, the ORs (95% CI) of NAFLD in Q2, Q3 and Q4 compared with Q1 were 0.88 (0.64–1.21), 0.71 (0.51–0.98) and 0.67 (0.48–0.95), respectively.

The association between Lp-PLA_2_ concentration and different NAFLD levels in was similar to the above results in Fig. 2. In model 1, the ORs (95% CI) for moderate to heavy NAFLD in Q2, Q3 and Q4 compared with Q1 were 0.71 (0.46–1.07), 0.49 (0.31–0.77) and 0.41 (0.25–0.66), respectively (P < 0.01). This relationship also persisted in models 2 and 3 even after adjusting for other potential confounders (Table 2). Our results indicated that increased Lp-PLA_2_ levels are associated with a decreased prevalence of NAFLD.

**Table 2:** Multivariate-adjusted OR and 95% CI for moderate and heavy NAFLD compared with light NAFLD at different Lp-PLA2 levels.

	OR (95% CI)		
	Model 1	Model 2	Model 3
Q1	Ref	Ref	Ref
Q2	0.71 (0.46–1.07)	0.74 (0.48–1.12)	0.64 (0.41–1.01)
Q3	0.49 (0.31–0.77)	0.54 (0.34–0.86)	0.48 (0.29–0.80)
Q4	0.41 (0.25–0.66)	0.46 (0.28–0.76)	0.47 (0.28–0.79)
*P* for trend	<0.01	<0.01	<0.01

OR: odd ratio; CI: confidence interval. The group of Q1 was regard as reference. Model 1: unadjusted. Model 2: adjusted for age and sex. Model 3: adjusted for age, sex, BMI, physical activity, education level, smoking, ALT, diabetes, hypertension and hyperlipidaemia.

## Discussion

In this present, large community-based study, we first demonstrated that higher Lp-PLA_2_ concentrations are associated with decreased NAFLD prevalence, and multivariate analysis revealed a significant trend over the Lp-PLA_2_ quartiles and a decreasing NAFLD OR gradient. In addition, a similar association was seen between the Lp-PLA_2_ quartiles and different NAFLD levels.

Our finding is inconsistent with a small sample sized study. A case-control analysis of 95 Turkish participants age around 40 years conducted by Yasar Colak revealed that Lp-PLA_2_ levels are higher in NAFLD patients than in healthy controls^13^. The study of Colak is a small sample size case-control study. Some unavoidable biases such as recall bias may exist in this type of retrospective study. Therefore, association between Lp-PLA_2_ and NAFLD was explored in our cohort study with a large sample size. Moreover, NAFLD is strongly associated with cardiovascular disease, hence we excluded participants suffering from cardiovascular diseases (stroke, coronary disease, transient ischemic attack). Therefore, the study population in the present study is a cardiovascular disease-free population, which might explain the inconsistency with Colak’s study. In addition, our participants were middle-aged and elder people (mean ages 61.6 ± 11.8) but subjects in Colak study were relative young. Hence, the relationship between Lp-PLA_2_ and NAFLD should be further explored.

However, several studies have also similar findings with us. Nozomu Kono, *et al*. indicated that Lp-PLA_2_ was protective against ox-LDL-induced hepatic injury, which is regarded as a leading cause of NAFLD^15,16^. In addition, Stanislav, *et al*. demonstrated that the serum Lp-PLA_2_ secretion was increased in response to endotoxin-induced hepatic damage. Increased Lp-PLA_2_ levels promoted the elimination of excess oxidized phospholipids to prevent further hepatic damage^17^. Meanwhile, Grypioti further determined that Lp-PLA_2_ inhibits oxidative stress-induced liver damage and promotes liver recovery through animal experiments^18^. These studies suggest that Lp-PLA_2_ has a positive effect on liver tissue protection. In addition, our results supported the idea that increased Lp-PLA_2_ levels are associated with decreased NAFLD prevalence.

We proposed several explanations to analyze the results in our study. First, serum Lp-PLA_2_ can hydrolyze oxidized phospholipids (oxPL), a part of ox-LDL, to decrease oxidative stress which has been demonstrated as a major leading cause of NAFLD^9,11,12,19–21^. Second, Lp-PLA_2_ may hydrolyze oxPL to increase paraoxonase (PON) expression, which is a beneficial enzyme for NAFLD^22,23^. Third, serum Lp-PLA_2_ may bind HDL to form HDL-Lp-PLA_2_; then, HDL-Lp-PLA_2_ exerts a positive regulatory effect on inflammation and dyslipidaemia to protect against NAFLD^24–27^. In addition, several studies have revealed the serum lipid regulatory effect of Lp-PLA_2_^24,25,28^, so our results that hyperlipidaemia significantly decreased with the elevation of Lp-PLA_2_ level in baseline further supported our finding. However, these proposed underlying mechanisms require further study.

To the best of our knowledge, previous studies have mainly focused on basic studies, not epidemiological studies, to show evidence that Lp-PLA_2_ plays a protective effect in the liver. Our study is the first epidemiological study especially indicates that Lp-PLA_2_ is beneficial for NAFLD in a large Chinese population. NAFLD is a worldwide health problem, particularly in subjects with middle or old age. Currently, diagnosis of NAFLD mainly depends on imaging examination and liver biopsy, therefore exploring a new effective serum biomarker is necessary for the diagnosis and prevention of NAFLD. Our finding that Lp-PLA_2_ associates with the initiation and progression of NAFLD might suggests that detection of Lp-PLA_2_ is helpful for the diagnosis and prevention of NAFLD.

Nevertheless, several limitations in our study must be noted. We were unable to measure the hepatic Lp-PLA_2_ expression, which is a better marker to study. Liver biopsies had the more accuracy to diagnose NAFLD than abdominal ultrasonography did, but it was with invasive property. So, abdominal ultrasonography was used in the current study for diagnosis of NAFLD. Besides, we did not measure Lp-PLA_2_ activity, which is more representative, but the Lp-PLA_2_ concentration is closely correlated with activity; thus, we selected concentration in our study. In addition, the present study was a cross-sectional study. These results need to be further investigated in a longitudinal design for testing the causality. Since the study is ongoing, more data are needed to further confirm the correlation between Lp-PLA_2_ and NAFLD in the future.

In conclusion, we have demonstrated that Lp-PLA_2_ is significantly associated with decreased NAFLD risk in a large Chinese population, and increased Lp-PLA_2_ levels may be useful as an independent protector against NAFLD.

## Methods

### Study population

**Figure 3:**
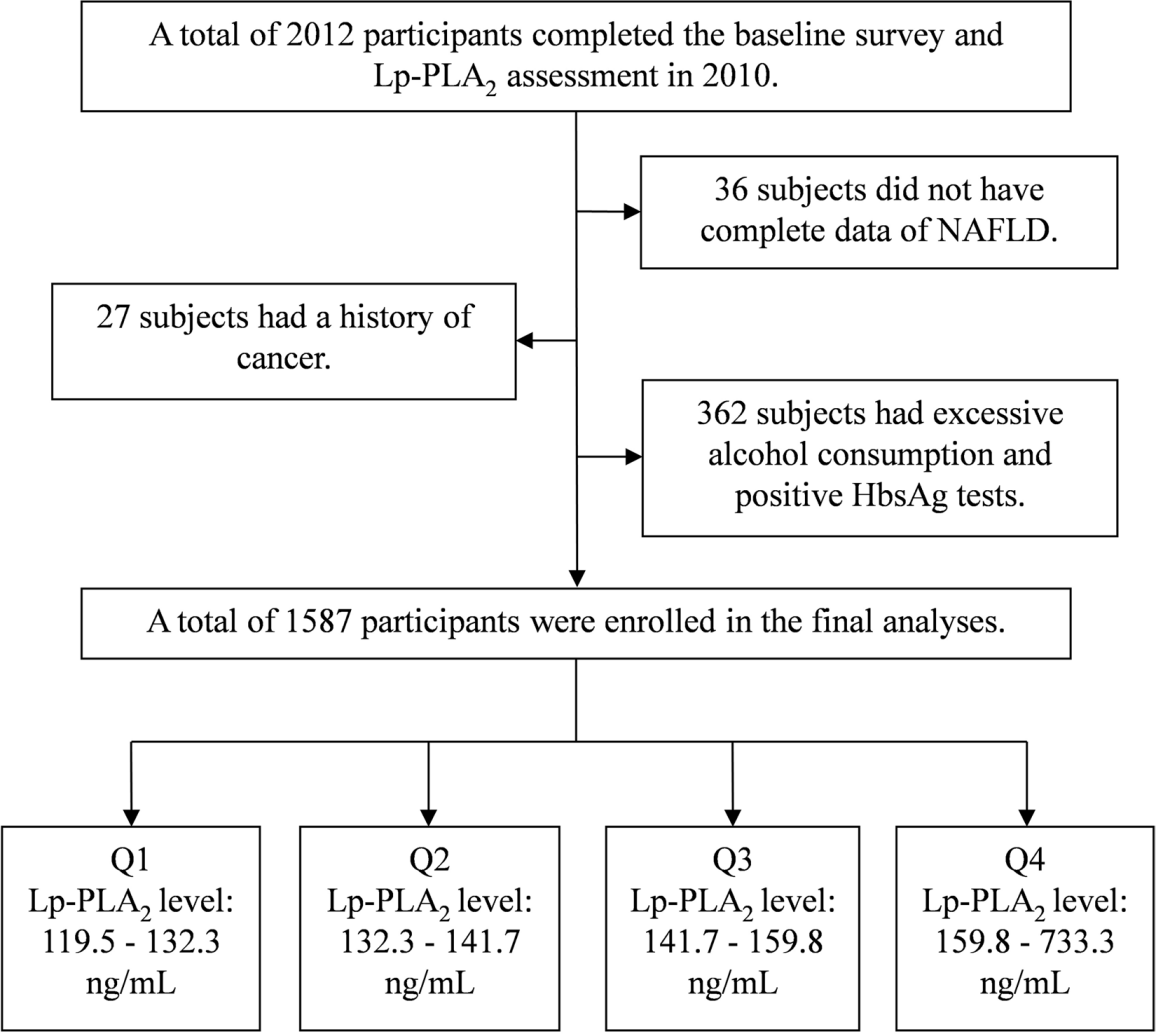
Flow chart of the study.

In the current study, we included 2012 participants ≥40 years old who had their serum Lp-PLA_2_ levels measured in the APAC (Asymptomatic Polyvascular Abnormalities in Community) study^29^. We further excluded the participants who had (1) no or incomplete NAFLD information (n = 36), (2) a history of cancer (n = 27), (3) excessive alcohol consumption (male ≥ 20 g/d, female ≥ 10 g/d) and a positive HbsAg test (n = 362). In total, 1587 individuals were included in this analysis (Fig. 3).

All participants underwent a standardized questionnaire survey, physical examinations and laboratory assessments conducted by 11 hospitals in the Kailuan community. The study was performed according to the Declaration of Helsinki guidelines and was approved by the Ethics Committees of Beijing Tiantan Hospital and Kailuan General Hospital. All subjects provided informed consent for participation in the study.

### Lp-PLA_2_ Assessment

Blood samples were drawn from the antecubital vein after an overnight fast and were collected in ethylene diamine tetraacetic acid (EDTA) tubes. The samples were stored at −80 °C for subsequent analyses after a centrifugation at least 10 min at 3000 r/min. The serum Lp-PLA_2_ concentration was measured by enzyme-linked immunosorbent assays (ELISAs) according to the manufacturer’s instructions^29^. The intra- and inter-assay coefficients of variation were <8% and <10%. To minimize the measurement error, the serum levels of Lp-PLA_2_ were also assessed by professional technicians in Beijing Tiantan Hospital simultaneously. The participants were classified into 4 groups according to the quartile of Lp-PLA_2_ concentration.

### NAFLD Assessment

NAFLD examination was performed via abdominal ultrasonography by two experienced radiologists who were blinded to laboratory participants. Fatty liver was diagnosed by a high-resolution B-mode topographic ultrasound system with a 3.5 MHz probe (ACUSON × 300, Siemens, Germany). The fatty liver diagnosis was based on the presence of at least two of the following three clinical findings: (1) diffusely increased liver near field ultrasound echo (‘bright liver’); (2) liver echo greater than kidney; (3) vascular blurring and the gradual attenuation of far field ultrasound echo. NAFLD was also categorized into three types: Light (5% < lipid content ≤ 10%), Moderate (10% < lipid content ≤ 25%), Heavy (25% < lipid content)^30,31^.

### Assessment of demographic variables and potential covariates

Participants reported basic information by answering questionnaires including age, sex, lifestyle behaviours and medical history. Smoking was categorized as none or current smoking on the basis of the self-report. Physical activity was evaluated from subject responses, and the subjects were categorized as inactive, moderate and active. Education level was classified as illiterate or primary, middle/high school or college and above.

All participants were measured by standing in light clothing without shoes and hats. Body weight was measured to the nearest 0.1 kg with the use of calibrated platform scales, and height was measured to the nearest 0.1 cm with the use of a tape ruler. Body mass index (BMI) was calculated as weight divided by the square of height.

Diseases history mainly included hypertension, diabetes and hyperlipidaemia. After a ≥5-min resting period, blood pressure was measured with a mercury sphygmomanometer twice at 5-min intervals on the left arm while the patient was in a seated position. The mean of 2 readings was used for analysis. A systolic blood pressure ≥140 mm Hg, diastolic blood pressure ≥90 mm Hg or a history of hypertension was defined as hypertension. Diabetes was defined as a fasting blood glucose concentration >7.0 mmol/L or a medical history of diabetes. The total cholesterol (TC) level was measured with the use of an enzymatic colorimetric method, and high-density lipoprotein cholesterol (HDL) and low-density lipoprotein cholesterol (LDL) levels were measured by direct tests (Mind Bioengineering Co. Ltd). Triglyceride (TG) and alanine aminotransferase (ALT) levels were also measured. Hyperlipidaemia was defined as a history of hyperlipidaemia, TC ≥ 5.7 mmol/L, TG ≥ 1.7 mmol/L, or the current use of lipid-lowering drugs. An autoanalyser (Hitachi 747; Hitachi) was used to analyse all plasma samples at the central laboratory of Kailuan General Hospital.

### Statistical analyses

Continuous variables are shown as the means ± standard deviation (ME ± SD) and were compared using an analysis of variance (ANOVA) or a t-test. Categorical variables are presented as frequencies (percentage) and were compared using the Chi-squared test. Multivariate logistic regression was used to evaluate the relationship between Lp-PLA2 concentration and NAFLD by calculating the odds ratio (OR) and 95% confidence interval (CI). Moreover, the association between Lp-PLA_2_ and different levels of NAFLD was analysed by logistic regression. Potential confounders such as age, sex, BMI, smoking, physical activity, education level, ALT, diabetes, hypertension and hyperlipidaemia were adjusted in statistical analyses. Statistical analysis was performed using SAS software, version 9.3 (SAS Institute Inc., Cary, NC, USA). P < 0.05 was regarded as significant for 2-sided tests.

## References

[1] Targher G, Marra F, Marchesini G (2008). Increased risk of cardiovascular disease in non-alcoholic fatty liver disease: causal effect or epiphenomenon. Diabetologia.

[2] Nugent C, Younossi ZM (2007). Evaluation and management of obesity-related nonalcoholic fatty liver disease. Nat Clin Pract Gastroenterol Hepatol.

[3] Chalasani N (2012). The diagnosis and management of non-alcoholic fatty liver disease: practice Guideline by the American Association for the Study of Liver Diseases, American College of Gastroenterology, and the American Gastroenterological Association. Hepatology.

[4] Chitturi S, Farrell GC (2007). Fatty liver now, diabetes and heart attack later? The liver as a barometer of metabolic health. J Gastroenterol Hepatol.

[5] Wu R (2017). Nonalcoholic Fatty Liver Disease and Coronary Artery Calcification in a Northern Chinese Population: a Cross Sectional Study. Sci Rep.

[6] Luo J (2015). Nonalcoholic fatty liver disease as a potential risk factor of cardiovascular disease. Eur J Gastroenterol Hepatol.

[7] Wang C, Fang X (2017). Lipoprotein-Associated Phospholipase A2 and Risk of Carotid Atherosclerosis and Cardiovascular Events in Community-Based Older Adults in China. Angiology.

[8] Garg PK (2017). Lipoprotein-associated phospholipase A2 and risk of incident peripheral arterial disease in a multi-ethnic cohort: The Multi-Ethnic Study of Atherosclerosis. Vasc Med.

[9] Abenavoli L, Milic N, Capasso F (2012). Anti-oxidant therapy in non-alcoholic fatty liver disease: the role of silymarin. Endocrine.

[10] Satapati S (2015). Mitochondrial metabolism mediates oxidative stress and inflammation in fatty liver. J Clin Invest.

[11] Matsuzawa A, Hattori K, Aoki J, Arai H, Inoue K (1997). Protection against oxidative stress-induced cell death by intracellular platelet-activating factor-acetylhydrolase II. J Biol Chem.

[12] Rosenson RS, Stafforini DM (2012). Modulation of oxidative stress, inflammation, and atherosclerosis by lipoprotein-associated phospholipase A2. J Lipid Res.

[13] Colak Y (2012). Association of serum lipoprotein-associated phospholipase A2 level with nonalcoholic fatty liver disease. Metab Syndr Relat Disord.

[14] Derbent A (2011). Serum platelet-activating factor acetylhydrolase activity: relationship with metabolic syndrome in women with history of gestational diabetes mellitus. Gynecol Endocrinol.

[15] Kono N (2008). Protection against oxidative stress-induced hepatic injury by intracellular type II platelet-activating factor acetylhydrolase by metabolism of oxidized phospholipids *in vivo*. Journal of Biological Chemistry.

[16] Kaikkonen JE (2016). Longitudinal study of circulating oxidized LDL and HDL and fatty liver: the Cardiovascular Risk in Young Finns Study. Free Radic Res.

[17] Svetlov SI, Sturm E, Olson MS, Crawford JM (1999). Hepatic regulation of platelet-activating factor acetylhydrolase and lecithin:cholesterol acyltransferase biliary and plasma output in rats exposed to bacterial lipopolysaccharide. Hepatology.

[18] Grypioti AD, Kostopanagiotou G, Mykoniatis M (2007). Platelet-activating factor inactivator (rPAF-AH) enhances liver’s recovery after paracetamol intoxication. Dig Dis Sci.

[19] Silva IT, Mello AP, Damasceno NR (2011). Antioxidant and inflammatory aspects of lipoprotein-associated phospholipase A2 (Lp-PLA2): a review. Lipids Health Dis.

[20] Tariq Z, Green CJ, Hodson L (2014). Are oxidative stress mechanisms the common denominator in the progression from hepatic steatosis towards non-alcoholic steatohepatitis (NASH). Liver Int.

[21] Gentric G (2015). Oxidative stress promotes pathologic polyploidization in nonalcoholic fatty liver disease. J Clin Invest.

[22] Van Lenten BJ, Wagner AC, Navab M, Fogelman AM (2001). Oxidized phospholipids induce changes in hepatic paraoxonase and ApoJ but not monocyte chemoattractant protein-1 via interleukin-6. J Biol Chem.

[23] Garcia-Heredia A (2013). Paraoxonase-1 deficiency is associated with severe liver steatosis in mice fed a high-fat high-cholesterol diet: a metabolomic approach. J Proteome Res.

[24] Tellis CC, Tselepis AD (2009). The role of lipoprotein-associated phospholipase A2 in atherosclerosis may depend on its lipoprotein carrier in plasma. Biochim Biophys Acta.

[25] Angeli V (2004). Dyslipidemia associated with atherosclerotic disease systemically alters dendritic cell mobilization. Immunity.

[26] Kim EJ (2014). Cholesterol-induced non-alcoholic fatty liver disease and atherosclerosis aggravated by systemic inflammation. Plos One.

[27] Katsiki N, Mikhailidis DP, Mantzoros CS (2016). Non-alcoholic fatty liver disease and dyslipidemia: An update. Metabolism.

[28] Tellis CC, Tselepis AD (2014). Pathophysiological role and clinical significance of lipoprotein-associated phospholipase A2 (Lp-PLA2) bound to LDL and HDL. Curr Pharm Des.

[29] Jiang R (2016). Higher Levels of Lipoprotein Associated Phospholipase A2 is associated with Increased Prevalence of Cognitive Impairment: the APAC Study. Sci Rep.

[30] Fan JG (2011). Guidelines for the diagnosis and management of nonalcoholic fatty liver disease: update 2010: (published in Chinese on Chinese Journal of Hepatology 2010, 18, 163–166). J Dig Dis.

[31] Farrell GC, Chitturi S, Lau GK, Sollano JD (2007). Guidelines for the assessment and management of non-alcoholic fatty liver disease in the Asia-Pacific region: executive summary. J Gastroenterol Hepatol.

